# An overview of the psychological effects of common contraceptive methods on women

**DOI:** 10.1192/j.eurpsy.2021.2211

**Published:** 2021-08-13

**Authors:** S. Haqshenas

**Affiliations:** Reproductive Health And Midwifery, Mazandaran University of Medical Sciences, Sari, Iran

**Keywords:** Contraception, psychological effects, women

## Abstract

**Introduction:**

The psychological effects of using any method of contraception are not hidden from anyone but in different methods, they have different effects.

**Objectives:**

We aim to investigate the different psychological effects of common contraceptive methods in women.

**Methods:**

A search conducted by keywords “contraception”, “psychological effects” and “women” in PubMed, Science Direct, Scopus and Clinical Key and Google Scholar search engine. Finally, data from 12 articles were used for this review study.

**Results:**

The positive and negative psychological effects were slightly different in consumers. The effect of OC and IUD and sterilization on sex life compared to condoms was reported to be positive and in menstrual experiences, OC consumers reported higher satisfaction than other methods, in particular, IUD. The regret in using sterilization was higher than in other methods. Psychopathological disorders and psychological disorders developed while using these methods should be differentiated. Negative psychological effects of women using contraceptive methods are often due to their mental background to a mother’s role and fertility and the conflict that exists in these methods with their mental image. Also, cooperation and understanding of spouses on the acceptance of these methods and their positive or negative impact has been reported to be very effective.

**Conclusions:**

Before providing any method of contraception, it is recommended to provide comprehensive counseling on each method and follow up with women while consuming to reduce these symptoms and improving their effectiveness.

**Disclosure:**

No significant relationships.

